# Consensus Virtual Screening Protocol Towards the Identification of Small Molecules Interacting with the Colchicine Binding Site of the Tubulin‐microtubule System

**DOI:** 10.1002/minf.202200166

**Published:** 2022-10-19

**Authors:** Edgar López‐López, Carlos M. Cerda‐García‐Rojas, José L. Medina‐Franco

**Affiliations:** ^1^ DIFACQUIM Research Group Department of Pharmacy School of Chemistry Universidad Nacional Autónoma de México Mexico City 04510 Mexico; ^2^ Departamento de Química y Programa de Posgrado en Farmacología Centro de Investigación y de Estudios Avanzados del Instituto Politécnico Nacional Mexico City 07000 Mexico

**Keywords:** ADMET, Anti-cancer, Cell-based assays, Drug discovery

## Abstract

Modification of the tubulin‐microtubule (Tub‐Mts) system has generated effective strategies for developing different treatments for cancer. A huge amount of clinical data about inhibitors of the tubulin‐microtubule system have supported and validated the studies on this pharmacological target. However, many tubulin‐microtubule inhibitors have been developed from representative and common scaffolds that cover a small region of the chemical space with limited structural innovation. The main goal of this study is to develop the first consensus virtual screening protocol for natural products (ligand‐ and structure‐based drug design methods) tuned for the identification of new potential inhibitors of the Tub‐Mts system. A combined strategy that involves molecular similarity, molecular docking, pharmacophore modeling, and *in silico* ADMET prediction has been employed to prioritize the selections of potential inhibitors of the Tub‐Mts system. Five compounds were selected and further studied using molecular dynamics and binding energy predictions to characterize their possible binding mechanisms. Their structures correspond to 5‐[2‐(4‐hydroxy‐3‐methoxyphenyl) ethyl]‐2,3‐dimethoxyphenol (**1**), 9,10‐dihydro‐3,4‐dimethoxy‐2,7‐phenanthrenediol (**2**), 2‐(3,4‐dimethoxyphenyl)‐5,7‐dihydroxy‐6‐methoxy‐4*H*‐1‐benzopyran‐4‐one (**3**), 13,14‐epoxyparvifoline‐4’,5’,6’‐trimethoxybenzoate (**4**), and phenylmethyl 6‐hydroxy‐2,3‐dimethoxybenzoate (**5**). Compounds **1–3** have been associated with literature reports that confirm their activity against several cancer cell lines, thus supporting the utility of this protocol.

## Graphical abstract

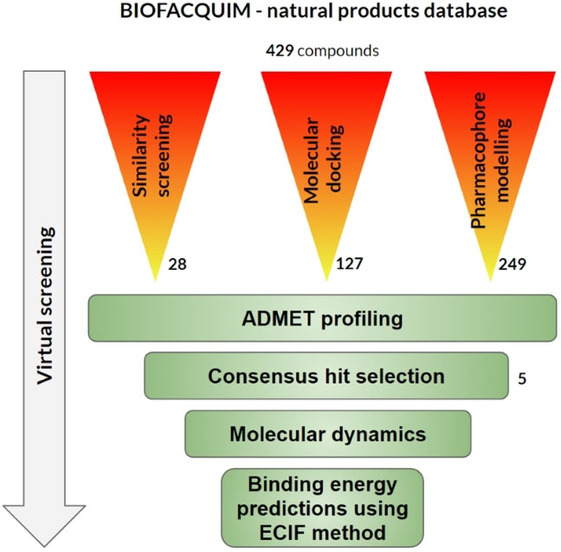

## 1 Introduction

The tubulin‐microtubule system (Tub‐Mts) is a key structural element of the cytoskeleton in eukaryotic cells and has an essential role in different cellular events including the formation of the centrosome (essential during the G_2_/M phase of the cell cycle), cell traffic, and cell motility. This complex machinery comprises a highly dynamic system since their elements constantly undergo polymerization and depolymerization processes. Interruption of this dynamic process can induce cell cycle arrest and lead to apoptosis. For this reason, the Tub‐Mts system is an attractive target for anticancer therapy.[^1^, ^2^] A definite number of binding sites have been reported that generate the modulation of the Tub‐Mts system, e. g., the most common colchicine, vinca, and paclitaxel binding sites.^[3]^ Depending on the site where the molecules interact with the Tub‐Mts system, they can be classified as microtubule stabilizers or destabilizers. The possibility of generating new small molecules that modulate the microtubules offers some advantages over the conventional therapeutic strategies, which include the possibility to reduce the high doses administrated in conventional cancer treatment (e. g., treatment with the paclitaxel) or increase the intracellular bioavailability, this could reduce side effects of the treatments.

Computational methods are demonstrated to be useful in the initial stages of the drug design and drug discovery process.^[4]^ In the context of the Tub‐Mts system, ligand‐based and structure‐based approaches, such as tridimensional quantitative structure‐activity relationships (3D‐QSAR), pharmacophore models, molecular docking, and molecular dynamics (MD),[^5^, ^6^] have been demonstrated their effectiveness for proposing novel Tub‐Mts inhibitors. However, the use of combined approaches (consensus approach) has shown to perform better than the isolated drug design methodologies.[^7^, ^8^]

There is a large diversity of compounds that interact directly with tubulin (inhibiting or promoting the polymerization of microtubules). However, the majority of potent inhibitors of the Tub‐Mts system show a poorly ADMET profile that has limited their clinical use.^[9]^ Accordingly, the development and implementation of virtual screening protocols with a consensus approach facilitates to propose novel inhibitors (against Tub‐Mts system) that besides could have an improved ADMET profile. Accordingly, in the present study, we explore the use of a consensus approach to propose novel natural products or derivatives with the potential as Tub‐Mts inhibitors. This protocol allows the prioritization of each potential candidate for prospective *in vitro* and *in vivo* tests.

## 2 Methodology

### 2.1 Construction of the In House Dataset

The data of 429 natural products and semi‐synthetic compounds isolated, characterized and/or designed in Mexico were stored in a database that is publicly available (BIOFACQUIM database).^[10]^ For each compound the canonical SMILES code was computed, which was used for prospective analyses.

### 2.2 Similarity Metrics

The similarity values of the 429 compounds from BIOFACQUIM dataset against each active reported compound to the Tub‐Mts system^[10]^ were computed using the MACCS keys (166‐bits) and ECFP4 fingerprints, which have been quantified using the Tanimoto coefficient. The molecular similarity analyses were generated in the KNIME software employing the RDKit node for molecular fingerprints generation and the CDK node for the similarity calculation.[^11^, ^12^]

### 2.3 Molecular Docking

The crystallographic structure of the α‐ and β‐tubulin (PDB ID: 4O2B) was obtained from the Protein Data Bank database.^[13]^ Water molecules and ligands were removed, while the hydrogens atoms and atom charges were assigned using the AMBER10_EHT forcefield implemented in the Molecular Operating Environment (MOE) software (v. 2021).^[14]^ All tested ligands (from BIOFACQUIM database) were constructed from their SMILES codes and were minimized using the MMFF94X forcefield.

The molecular docking was generated using the Triangle Matched Pair method and the GBVI/WSA scoring function as implemented in the MOE software. The residues around the binding site of colchicine (4.5×4.5×4.5 Å) were explored. For each ligand, 30 induced fit conformational searches were generated, and the conformations with better binding scores were selected for further studies (screening with the pharmacophore model and molecular dynamics).

### 2.4 Pharmacophore Modeling

The binding modes (calculated using molecular docking) of the 20 most active compounds with different Bemis‐Murcko scaffold, that have been associated with the interaction of the colchicine binding site and the modulation of the Tub‐Mts system, that additionally have been associated with micromolar/nanomolar activity against cancer cell lines, were used to construct the pharmacophore model.^[4]^ Each pharmacophoric features were selected automatically (consider that are present in at least 70 % of the reference compounds; i. e., features present in 14 of 20 most active and diverse compounds) using the module *pharmacophore* implemented in MOE software. Figure S1 in the Supporting Information illustrates the model used in this protocol. In contrast with previous reports, this pharmacophore model is inspired on the huge diversity of scaffolds reported as inhibitors, and not in a large quantity of compounds of a unique analog series (or compounds structurally related). Namely, the novelty of this pharmacophoric model is that it uses different binding modes of different reported potent scaffolds.[^15^, ^16^, ^17^]

### 2.5 ADME/Tox Profiling

Pharmacokinetics (e. g., gastrointestinal absorption, inhibition of cytochromes, etc.) and the medicinal chemistry profile (e. g., PAINS ‐ pan‐assay interference compounds) were computed for the 429 compounds (contained on the BIOFACQUIM database) using the SwissADME server (http://www.swissadme.ch/index.php).^[18]^

### 2.6 Virtual Screening

The 429 compounds contained in the BIOFACQUIM database were screened and selected using different ligand‐ and structure‐based criteria: (1) compounds with higher similarity values with respect to at least one reported active compound; (2) compounds with a better binding score; (3) compounds with at least five (of six) key pharmacophoric features; (4) compounds with desirable drug‐likeness, solubility, and cytochromes′ inhibition properties. Compounds with the four criteria were selected for prospective MD studies.

### 2.7 Molecular Dynamics

MD studies of the protein‐ligand complexes were performed using Desmond (version 2021–1, Schrödinger, New York, NY, USA) with the OPLS 2005 forcefield.^[19]^ The most representative docking pose for each ligand was used as a starting point to initiate the MD simulations. The complexes were prepared with the System Builder Utility in a buffered orthotopic box (10×10×10 Å), using the transferable intermolecular potential with a 3‐point model for water (TIP3P). The complexes were neutralized and NaCl was added in a 0.15 M concentration. Complexes were minimized using the steep‐descent (SD) algorithm followed by the Broyden‐Fletcher‐Goldfarb‐Shanno (LBFGS) method in three stages. In the first stage, water‐heavy atoms were restrained with a force constant of 1000 kcal mol^−1^ Å^−2^ for 5000 steps (1000 SD, 4000 LBFGS) with a convergence criterion of 50 kcal mol^−1^ Å^−2^; for the second stage, backbones were constrained with a 10 kcal mol^−1^ Å^−2^ force constant using a convergence criterion of 10 kcal mol^−1^ Å^−2^ for 2000 steps (1000 SD, 1000 LBFGS); and for the third stage, the systems were minimized with no restraints for 1000 steps (750 SD, 250 LBFGS) with a convergence criterion of 1 kcal mol^−1^ Å^−2^. Equilibration was carried out in several steps, beginning with Brownian Dynamics for 250 ps with the Berendsen thermostat, followed by simulation on the NVT ensemble, slowly heating from 10 to 300 K over 3000 ps. At this stage, constraints were enforced on solute heavy atoms, using a constant of 50 kcal/mol. Finally, equilibration on the NPT ensemble used the Berendsen thermostat and Langevin barostat for additional 250 ps. Subsequently, the system was submitted to 120 ns of production runs, under NPT ensemble at 1 bar using the Martyna‐Tuckerman‐Klein (MTK) barostat and 300 K using the Nosé–Hoover thermostat. Electrostatic forces were calculated with the smooth PME method using a 9 Å cut‐off, while constraints were enforced with the M‐SHAKE algorithm. Integration was done every 1.2 fs, with a recording interval of 50 ps. The quality of the simulations was carried out with the tools implemented in the Maestro‐GUI (Schrödinger, New York, NY, USA) (Figure S2 in the Supporting Information).

### 2.8 Binding Energy Prediction

Extended connectivity interaction features (ECIF) are an open tool based on machine learning approaches that predict the binding energy (*p*Ki) of a protein‐ligand complex. ECIF use the interaction generated between the ligand and complex (e. g., tubulin‐inhibitor complex) to predict their affinity.^[20]^ The final MD binding modes (available on the Supporting Information) of the selected compounds were used to generate the binding energy predictions. The code to calculate the binding energy predictions is freely available at https://github.com/DIFACQUIM/ECIF.

## 3 Results and Discussion

**Figure 1:**
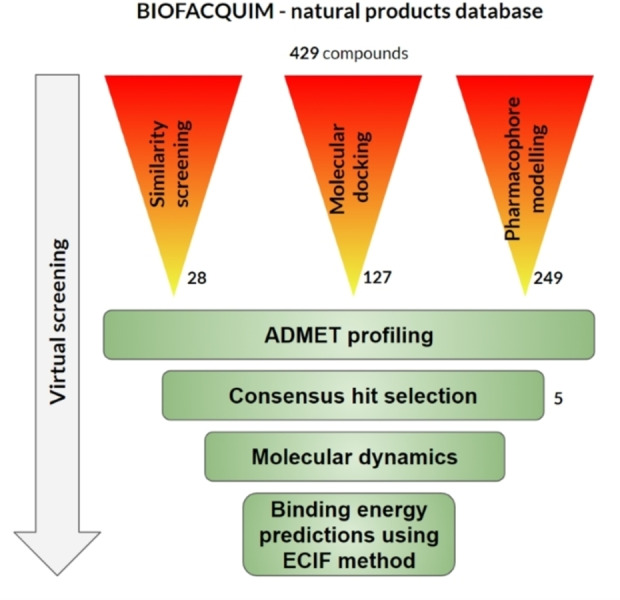
Workflow of the virtual screening approach for the identification of potential Tub‐Mts inhibitors. As part of the methodology, the recently developed ECIF was implemented.^[20]^

This paper describes the development and the results of a consensus virtual screening protocol (i. e., ligand‐ and structure‐based approaches) followed by the combined analysis to select hit compounds for prospective experimental testing as inhibitors of the Tub‐Mts system. Figure 1 shows an overview of the virtual screening protocol.

### 3.1 Similarity Screening

From 429 analyzed compounds only 28 (6.5 %) were considered hits. Namely, 28 compounds have been associated with consensus similarity values above the median plus one standard deviation for almost one reported active compound (0.806 and 0.314, respectively for MACCS keys and ECFP4 fingerprints).

### 3.2 Molecular Docking

**Figure 2:**
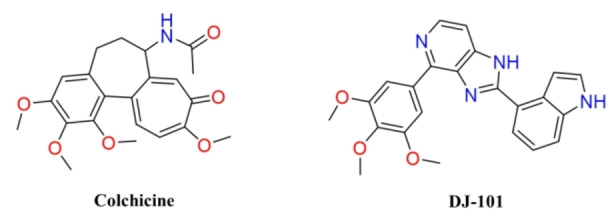
Reference compounds used in docking.

Molecular docking was performed using MOE (PDB ID: 4O2B). The docking score for the reference compounds (Figure 2) was −10.01 (colchicine) and −10.24 (DJ‐101). Only 37 of 429 (8 %) tested compounds were considered tentative hits (i. e., 37 compounds show better binding score than colchicine). However, the diverse size of the tested compounds (from 122.12 up to 2501.11 g/mol) could be generating false negatives. Accordingly, we normalize the binding score using the number of non‐hydrogen atoms (binding score/# non‐H atoms), obtaining 127 (29 %) selected hits (values equal to or lower than −0.345, adapting the binding scoring value to colchicine). The controls (colchicine and DJ‐101) and the selected compounds had predicted interactions with almost three of the reported key interactions of colchicine (Ala 180, Val 181, Cys 241, Met 259, and Ala 316) and/or the reported key interactions of DJ‐101 (Val 181, Val 236, Leu 246, Asn 247, Asn 347, and Lys 350).[^21^, ^22^, ^23^]

### 3.3 Pharmacophore Based Screening

The pharmacophore model of colchicine binding site inhibitors (Figure S1) was used to identify compounds with key pharmacophoric features associated to inhibition of tubulin. For example, the generation of H‐donor and acceptor bonding, aromatics centers, and hydrophobic regions as illustrated in Figure S1. Pharmacophore‐based screening identified 249 of 429 compounds in the screening data set that gather all six pharmacophoric key features. Thus, close to 58 % of the virtually screened compounds with the pharmacophore‐based approach exhibit a similar binding mode to that of reported active compounds.^[9]^

### 3.4 ADMET Profile

For all studied compounds, their ADMETox‐related descriptors were computed with SwissADME. As described hereunder, some of these descriptors were used as a guide for the prioritization of hit compounds. The main types of considered ADMETox descriptors were those associated with drug‐likeness, solubility, and cytochromes inhibition.

### 3.5 Hit Selection

**Figure 3:**
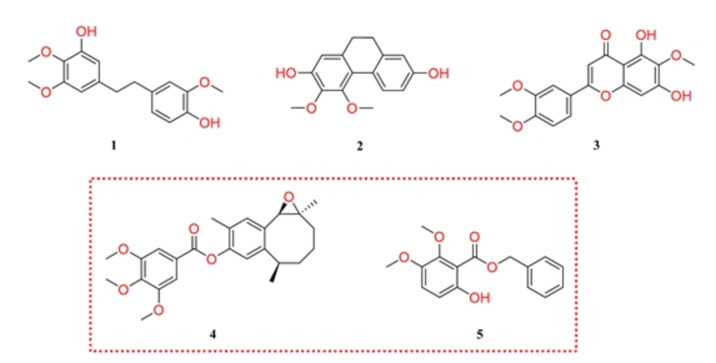
Potential inhibitors of the Tub‐Mts system identified by the virtual screening protocol. From BIOFACQUIM database, only five compounds fully met the selection criteria raised in the virtual screening protocol. The red dotted box remarks the compounds without previous activity reports against cancer cell lines.

The consensus virtual screening protocol allowed the prioritization of potentially active compounds for the Tub‐Mts system. In this case, the 429 compounds were screened from which 127 met the initial inclusion criteria (higher similarity than the active compounds,^[9]^ equal or lower binding score than colchicine, and similar binding modes to the reported active compounds). However, we prioritize the selection of the five most potential inhibitors according to the diversity of their scaffolds (i. e., we selected the most potent compounds with different Bemis‐Murcko scaffold to explore the major possible amplitude of chemical space on the BIOFACQUIM dataset) and their ADMET *in silico* profile. The structures of these compounds are shown in Figure 3. The complete list of screened compounds and their consensus parameters are available in the Supporting Information (Table S1).

Three (**1**–**3**) of the five selected compounds have shown inhibitory activity against different cancer cell lines. Thus, 5‐[2‐(4‐hydroxy‐3‐methoxyphenyl)ethyl]‐2,3‐dimethoxyphenol (**1**) (Figure 3), identified as FQNP278 in Table S1 in the Supporting Information, exhibits cytotoxic activity against the HL60 (IC_50_: 24 μM) and MCF7 (IC_50_: 27 μM) cell lines,^[24]^ 9,10‐dihydro‐3,4‐dimethoxy‐2,7‐phenanthrenediol or erianthridin (**2**), identified as FQNP280 in Table S1, exhibits cytotoxic activity against the HL60 (IC_50_: 44 μM), H460 (IC_50_: 150 μM), A549 (IC_50_: 161 μM) cell lines,[^25^, ^26^] 2‐(3,4‐dimethoxyphenyl)‐5,7‐dihydroxy‐6‐methoxy‐4*H*‐1‐benzopyran‐4‐one (**3**), identified as FQNP112 in Table S1, shows cytotoxic activity against the MKN1 (IC_50_: 50 μM), SNU601 (IC_50_: 50 μM), and MCF7 (IC_50_: 90 μM) cell lines as well as cytostatic activity against NCI‐60 (GI_50_: 15 μM) cell line.[^27^, ^28^] Additionally, compound **3** has been tested on tubulin polymerization assays showing inhibitory activity (IC_50_:>40 μM).^[29]^

Interestingly, the administration of compounds **1**–**3** on different cancer cell lines regulates their apoptosis events by the activation of Bcl‐2 and caspase 3 pathway,[^22^, ^23^, ^24^, ^25^] these molecular events are the same observed when Tub‐Mts system inhibitors (e. g. colchicine) are administrated.[^1^, ^2^] Therefore, the activity reported for compound **1**–**3** could be explained (at least in part) by their inhibition of the Tub‐Mts system.

**Figure 4:**
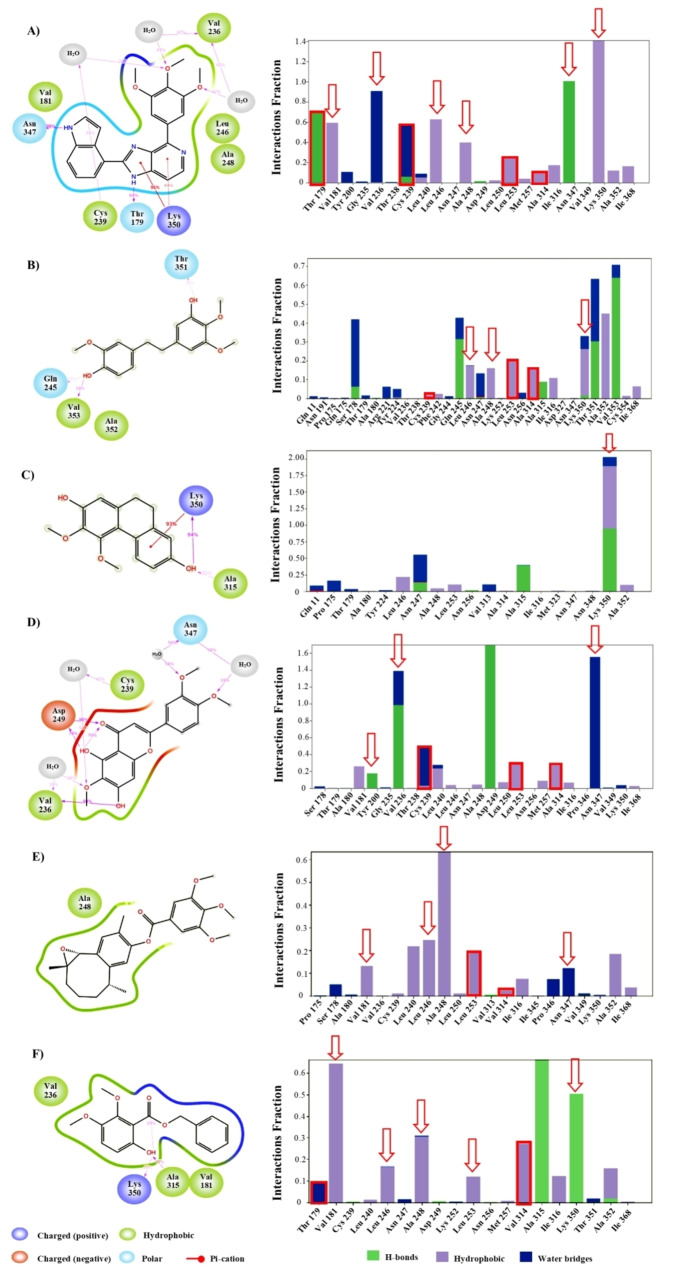
Key interactions during the molecular dynamics. Molecular dynamics results of (A) DJ‐101 (control); (B) compound **1**; (C) compound **2**; (D) compound **3**; (E) compound **4**; and (F) compound **5**. Compounds in panels B–F have been selected according to the virtual screening results. The red boxes remark the key interactions reported for colchicine (classical inhibitor). Additionally, the red arrows show the key interactions reported for DJ‐101 (novel inhibitor).

Compounds **1**–**3**, as well as those compounds without activity reports, 13,14‐epoxyparvifoline‐4’,5’,6’‐trimethoxybenzoate (**4**), and phenylmethyl 6‐hydroxy‐2,3‐dimethoxybenzoate (**5**) were further studied herein using MD to explore their binding modes with tubulin (Figure 4).

DJ‐101 and colchicine were used as control compounds in this protocol. DJ‐101 is a potent inhibitor (7–10 nM) on several cell lines whose activity has been associated with the inhibition of the Tub‐Mts system. Their interactions on the colchicine binding site have been characterized using crystallographic approaches.^[30]^ Figure 4A shows the MD results of DJ‐101 (120 ns of production), that are in agreement with its previous crystallographic report, showing that the compound interacts with key residues such as Thr 179, Val 181, Val 236, Cys 239, Leu 246, Ala 248, Asn 347, and Lys 350. Compound **1** shows predictive eleven MD interactions (Figure 4B) with the colchicine binding site, of which five are considered key interactions (Leu 246, Ala 248, Leu 253, Ala 314, and Lys 350), while compound **2** (Figure 4C) showed one interaction (Lys 350) during the MD. In contrast, compound **3** showed nine MD interactions, while compounds **4** and **5** exhibit ten interaction (during MD simulations) each. Interestingly, compounds **3** and **5** matched two key interactions with respect to DJ‐101, Val 236, and Asn 347 for **3** (Figure 4D), and Val 181 and Lys 350 for **5** (Figure 3F), while compound **4** matched one key interaction from the MD simulations (Ala 248, Figure 4E).

### 3.6 Limitations of the Virtual Screening Protocol

The huge diversity of conformational states in the Tub‐Mts system generates limitations in the available structure‐based design methods (e. g., docking, pharmacophoric modeling, and MD). This diversity could arise from different isoforms of tubulin and their interaction with other associated proteins. Also, the plethora of druggable cavities and ligand binding modes make it difficult to use these methods alone. Additionally, the structure‐based methods used in this protocol do not consider aspects related to the drug resistance associated with the transport of glycoprotein‐P or the amino acid mutations on the different isoforms of tubulins. In this sense, the combination of several complementary approaches including similarity searching and prediction of ADMET properties, also included in this protocol, has demonstrated the dramatic reduction of false‐positive cases.[^31^, ^32^, ^33^, ^34^]

Finally, it is important to remark that the inhibitory potential of selected compounds in this virtual screening protocol will need to be confirmed using polymerization tubulin assays, crystallographic approaches, and cancer cell‐based assays.

## 4 Conclusions

Herein, we report a consensus (structure‐ and ligand‐based) virtual screening protocol of the BIOFACQUIM chemical database, namely, 429 compounds (natural products and semi‐synthetic ligands) that were tested to identify potential drug candidates for the inhibition of the Tub‐Mts system. This protocol allowed the identification of five potential inhibitors which are 5‐[2‐(4‐hydroxy‐3‐methoxyphenyl)ethyl]‐2,3‐dimethoxyphenol (**1**), 9,10‐dihydro‐3,4‐dimethoxy‐2,7‐phenanthrenediol (erianthridin) (**2**), 2‐(3,4‐dimethoxyphenyl)‐5,7‐dihydroxy‐6‐methoxy‐4*H*‐1‐benzopyran‐4‐one (eupatilin) (**3**), 13,14‐epoxyparvifoline‐4’,5’,6’‐trimethoxybenzoate (**4**), and phenylmethyl 6‐hydroxy‐2,3‐dimethoxybenzoate (**5**). Compounds **1**–**3** have been associated with literature reports that confirm their activity against different cancer cell lines (IC_50_ values from 24 μM to 151 μM). Additionally, these compounds (**1**–**3**) modulate the apoptotic events by the activation of Bcl‐2 and caspase 3 pathway, these molecular events are the same observed when Tub‐Mts system inhibitors (e. g. colchicine) are administrated. The similarity metrics and molecular docking has been more rigorous with respect to the pharmacophore model for screening new potential compounds. However, each methodology contributes to select compounds with the major inhibitory potential of the Tub‐Mts system.

As a perspective, we will evaluate the selected compounds in tubulin polymerization inhibition and cell growth inhibition assays to confirm the inhibitory potential of these compounds. Additionally, we will evaluate this protocol using other natural products databases and small molecule libraries with the goal to explore and expand the chemical space of Tub‐Mts inhibitors. Finally, we remark that the binding energy predictions (using the ECIF tool) could be used as a new benchmark for future virtual screening protocols.

## Supporting Information

Pharmacophore model of active compounds against the Tub‐Mts system (Figure S1), Molecular dynamics quality results (Figure S2), Complete list of screened compounds and their consensus parameters (Table S1), The final molecular dynamics binding modes of the selected compounds were used to generate the binding energy predictions (File_S1.rar). List of screened compounds and their consensus parameters (Table S1).

## Conflict of Interest

None declared.

5

## Supporting information

As a service to our authors and readers, this journal provides supporting information supplied by the authors. Such materials are peer reviewed and may be re‐organized for online delivery, but are not copy‐edited or typeset. Technical support issues arising from supporting information (other than missing files) should be addressed to the authors.

Supporting Information

## Data Availability

The data that supports the findings of this study are available in the supplementary material of this article.
