# Supplementary material for: Consensus Virtual Screening Protocol Towards the Identification of Small Molecules Interacting with the Colchicine Binding Site of the Tubulin‐microtubule System
Source: Mol Inform. 2022 Oct 19;42(1):2200166. doi: 10.1002/minf.202200166 (PMC10078098; doi:10.1002/minf.202200166)
Supplement: Supplementary file 1 — Supporting Information [file MINF-42-0-s001.pdf]

molecular informatics

Supporting Information

Consensus Virtual Screening Protocol Towards the Identification of Small Molecules Interacting with the Colchicine Binding Site of the Tubulin-microtubule System

Edgar López-López, Carlos M. Cerda-García-Rojas, and José L. Medina-Franco*This is an open access article under the terms of the Creative Commons Attribution License, which permits use, distribution and reproduction in any medium, provided the original work is properly cited.

Supporting Information

Consensus Virtual Screening Protocol Towards the Identification of Small Molecules Interacting with the Colchicine Binding Site of the Tubulin-Microtubule System

Edgar López-López,[a, b] Carlos M. Cerda-García-Rojas,[b] and José L. Medina-Franco*[a]

[a] *DIFACQUIM Research Group, Department of Pharmacy, School of Chemistry, Universidad Nacional Autónoma de México, Mexico City 04510, Mexico*
E-mail: medinajl@unam.mx; Tel.: +52-55-5622-3899

[b] *Departamento de Química y Programa de Posgrado en Farmacología, Centro de Investigación y de Estudios Avanzados del Instituto Politécnico Nacional, Mexico City 07000, Mexico*

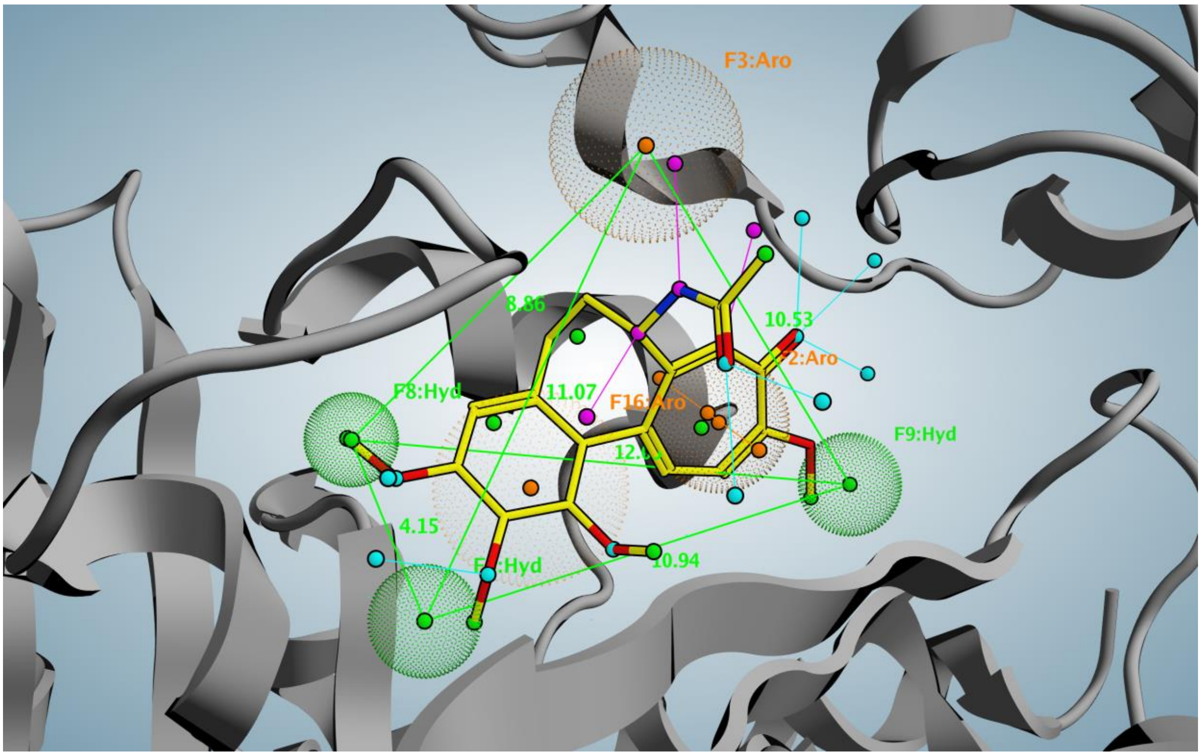

Figure S1. Pharmacophore model of active compounds against the Tub-Mts system that interact with the colchicine binding site. The colchicine is illustrated in yellow. Different pharmacophoric regions are distinguished using different colors, for example, pink (donor hydrogens) cyan (acceptor hydrogens), orange (aromatic interactions), and green (hydrophobic interactions), which represent the key interactions of the Tub-Mts inhibitors.

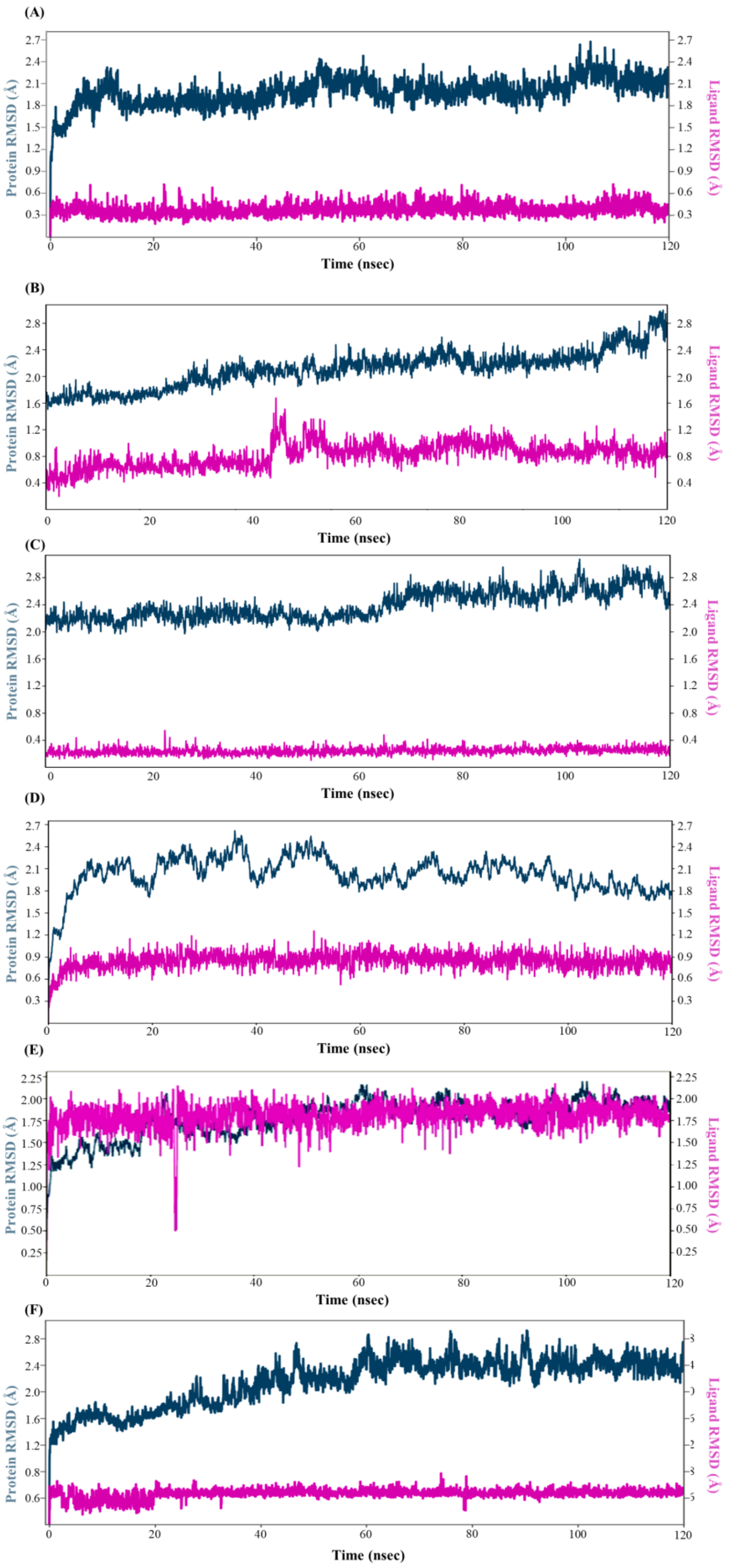

Figure S2. Molecular dynamics quality results (A) of DJ-101 (control), (B) of (**1**), (C) of (**2**), (D) of (**3**), (E) of (**4**), and (F) of (**5**).
